# The Characterization of Scaffolds Based on Dialdehyde Chitosan/Hyaluronic Acid

**DOI:** 10.3390/ma14174993

**Published:** 2021-09-01

**Authors:** Sylwia Grabska-Zielińska, Adrianna Sosik, Anna Małkowska, Ewa Olewnik-Kruszkowska, Kerstin Steinbrink, Konrad Kleszczyński, Beata Kaczmarek-Szczepańska

**Affiliations:** 1 Department of Physical Chemistry and Physicochemistry of Polymers, Faculty of Chemistry, Nicolaus Copernicus University, Gagarin 7, 87-100 Toruń, Poland; olewnik@umk.pl; 2 Department of Biomaterials and Cosmetics Chemistry, Faculty of Chemistry, Nicolaus Copernicus University, Gagarin 7, 87-100 Toruń, Poland; 291031@stud.umk.pl (A.S.); 291020@stud.umk.pl (A.M.); beata.kaczmarek@umk.pl (B.K.-S.); 3 Department of Dermatology, University of Münster, Von-Esmarch-Str. 58, 48149 Münster, Germany; kerstin.steinbrink@ukmuenster.de (K.S.); konrad.kleszczynski@ukmuenster.de (K.K.)

**Keywords:** dialdehyde chitosan, hyaluronic acid, scaffolds, tissue engineering

## Abstract

In this work, two-component dialdehyde chitosan/hyaluronic acid scaffolds were developed and characterized. Dialdehyde chitosan was obtained by one-step synthesis with chitosan and sodium periodate. Three-dimensional scaffolds were prepared by the lyophilization method. Fourier transform infrared spectroscopy (FTIR) was used to observe the chemical structure of scaffolds and scanning electron microscopy (SEM) imaging was done to assess the microstructure of resultant materials. Thermal analysis, mechanical properties measurements, density, porosity and water content measurements were used to characterize physicochemical properties of dialdehyde chitosan/hyaluronic acid 3D materials. Additionally, human epidermal keratinocytes (NHEK), dermal fibroblasts (NHDF) and human melanoma cells (A375 and G-361) were used to evaluate cell viability in the presence of subjected scaffolds. It was found that scaffolds were characterized by a porous structure with interconnected pores. The scaffold composition has an influence on physicochemical properties, such as mechanical strength, thermal resistance, porosity and water content. There were no significant differences between cell viability proliferation of all scaffolds, and this observation was visible for all subjected cell lines.

## 1. Introduction

Polysaccharides exist as homo- and copolymers, and they have been applied in packaging, wound dressings, cosmetics, etc. Most polysaccharides possess a linear structure, but there is also a branched structure with glycosidic bonds linking the basic structural units. For instance, among the compounds that belong to the most abundant polysaccharides in nature is cellulose. Chitin or chitosan are the main components of the shells of crustaceans, the outer skeletons of insects and starch occurring in plants, i.e., corn, potatoes or wheat [1,2,3].

Hyaluronic acid (HA) is a polysaccharide of unsulfated glycosaminoglycan (GAG) present in the extracellular matrix (ECM) of many soft connective tissues. It consists of alternating units of D-glucuronic acid and N-acetyl-D-glucosamine, interconnected alternately by β-1,4 and β-1,3 glycosidic bonds [4]. It is a biocompatible, biodegradable, bioactive, non-immunogenic and non-thrombogenic material [5]. Therefore, widespread applications within biomaterials are commonly reported. Materials based on hyaluronic acid are obtained by chemical modifications to ensure their mechanical and chemical strength. Derivatives of HA have physicochemical properties that can differ significantly from the native polymer, but most of the derivatives retain the biocompatibility and biodegradability of native HA. The stability of hyaluronic acid-based hydrogels depends on their resistance to degradation by hyaluronidases, as well as reactive oxygen and nitrogen species [6].

A simple method to improve the physicochemical properties of hyaluronic acid-based materials is to mix hyaluronic acid with other biopolymers, because one component material can be characterized by low physicochemical parameters, e.g., mechanical properties, water stability, density or porosity. These parameters may not be sufficient for biomedical applications. Therefore, scientists from all over the world mix biopolymers to obtain two- or three-component composites that would have improved properties in comparison with single-component composites (e.g., pure hyaluronic acid). It is well documented that hyaluronic acid is successfully mixed with other polysaccharides such as chitosan [7], alginate [8] or chondroitin sulphate [9]. Additionally, it may be modified by the protein supplementation, e.g., gelatin [10], collagen [11] or silk fibroin [12]. 

Dialdehyde chitosan is a relatively new substance. The difference between chitosan and dialdehyde chitosan is that during the process of periodate oxidation, chitosan receives multiple functional aldehyde groups which can easily and quickly react with functional groups from other polymers or cross-linking agents. It was reported that dialdehyde chitosan was used to cross-link collagen materials [13], chitosan films [14], cotton fabrics [15] and silk fibroin/collagen/chitosan scaffolds [16]. Dialdehyde chitosan was used as a substance to improve physicochemical properties of hyaluronic acid-based materials. Herein, the aim of our study was to obtain scaffolds based on a dialdehyde chitosan/hyaluronic acid mixture as a novel method of hyaluronic acid-based materials modification. In our opinion, hyaluronic acid is a better candidate to use in skin tissue engineering than collagen, because of its hygroscopic character, and its ability to swell and absorb a large amount of water. Moreover, dialdehyde chitosan was used to improve physicochemical properties of native hyaluronic acid materials. Our study focuses on the characterization of dialdehyde chitosan/hyaluronic acid-loaded scaffolds to be used in biomedical applications, and especially for tissue regeneration purposes [17,18]. 

## 2. Materials and Methods

### 2.1. Materials

Reagents purchased from Sigma-Aldrich (St. Louis, MO, USA) included: hyaluronic acid (M_v_ = 1.8 × 10^6^ g/mol, chitosan (DD = 78%; M_v_ = 1.4 × 10^6^ g/mol), acetic acid, acetone, sodium periodate and hydrochloric acid. Dialdehyde chitosan was obtained by one-step synthesis according to Bam et al. [13] with slight modifications introduced by Węgrzynowska-Drzymalska et al. [14], while the synthesis of the dialdehyde chitosan itself was detailed in our previous report [16].

### 2.2. Samples Preparation

Dialdehyde chitosan and hyaluronic acid were dissolved separately in water at 1% concentration. Subsequently, the substances were mixed in different ratios (*w*/*w*) as follows: 90/10, 80/20, 70/30, 60/40 and 50/50, and the resultant solutions were homogenized on the magnetic stirrer for 1 h. Next, the mixtures were poured into 24-well polystyrene culture plates, frozen and lyophilized (ALPHA 1–2 LDplus, CHRIST, −20 °C, 100 Pa, 48 h). The obtained scaffolds were evaluated as described below; however, the scaffold based on the mixture 90/10 was not considered during the experiment as it was soft, and the solid-state of the scaffold was not obtained.

### 2.3. Fourier Transform Infrared Spectroscopy—Attenuated Total Reflectance (FTIR–ATR)

Nicolet iS10 spectrophotometer equipped with an attenuated total reflectance (FTIR-ATR) device with a germanium crystal (Nicolet iS10, Thermo Fisher Scientific, Waltham, MA, USA) was used to assess the chemical structure of the obtained scaffolds. The spectra were evaluated in the range of 600–4000 cm^−1^. All spectra were recorded with the resolution of 4 cm^−1^ with 64 scans. Spectra were recorded for freeze-dried 3D materials.

### 2.4. Scanning Electron Microscopy (SEM)

The morphology of the samples was studied using a scanning electron microscope (SEM) (LEO Electron Microscopy Ltd., Cambridge, UK). Scaffolds were cut using the razor and covered with gold for further observation.

### 2.5. Thermal Properties

Thermogravimetric analysis and differential thermal analysis (TG-DTA) was performed at a heating rate of 10 °C/min (20–750 °C) in a nitrogen atmosphere by using TA Instruments SDT 2960 Simultaneous TGA-DTA (TA Instruments manufacturer, Eschborn, Germany). From the thermogravimetric curves, the characteristic temperature at a maximum decomposition rate of the investigated composites was determined.

### 2.6. Mechanical Properties

Mechanical properties were measured with the use of a mechanical testing machine (Shimadzu EZ-Test EZ-SX, Shimadzu, Kyoto, Japan). Scaffolds (diameter: 20 mm, height: 13 mm) were introduced between two discs and compressed (the starting speed of 200 mm min^−1^; initial force of 0.1 N; speed of the compression of 5 mm min^−1^). Parameters were selected based on the principal studies. The compressive modulus (Young modulus for the compression process), the maximum tension and the percentage deformation at maximum tension were determined with the Trapezium X Texture program. The compressive modulus was calculated from the linear region on the stress strain curves (0.05–0.15 kPa). The statistical analyses were made using a One-way ANOVA test.

### 2.7. Density, Porosity and Water Content

The liquid displacement method with isopropanol was used to measure the density and porosity of the scaffolds. A fragment of the sample with a known weight was immersed in a cylinder with a known volume of isopropanol for 3 min. The density was calculated using Equation (1):

(1) $$d\;\left[\frac{\mathrm{mg}}{{\mathrm{cm}}^{3}}\right]=\frac{W}{V_{2}-V_{3}}\cdot 100\%$$

where:

*W*—weight of sample [mg],

*V*_2_—total volume of isopropanol with the isopropanol impregnated sample [cm^3^],

*V*_3_—volume of isopropanol after scaffold removal [cm^3^].

The porosity was calculated using Equation (2):

(2) $$\varepsilon \;\left[\%\right]=\frac{V_{1}-V_{3}}{V_{2}-V_{3}}\times 100\%$$

where:

*V*_1_—initial volume of isopropanol [cm^3^],

*V*_2_*, V*_3_—as above.

The water content of the scaffolds was measured by drying samples at 105 °C until they reached a constant weight. The results were expressed as grams of water per 100 g of a dry sample [19]. For each kind of material, three samples were measured (*n* = 3).

### 2.8. Cell Culture

Normal human epidermal keratinocytes (NHEK) and normal human dermal fibroblasts (NHDF) were supplied by PromoCell (Heidelberg, Germany) and American Type Culture Collection (ATCC) (Manassas, VA, USA), respectively. NHEK were grown in Keratinocyte Growth Medium 2 supplemented with 1% penicillin-streptomycin solution, while NHDF were maintained in an MEM medium supplemented with 10% (*v*/*v*) heat-inactivated fetal bovine serum, 2 mM L-glutamine and 1% (*v*/*v*) streptomycin-penicillin solution. Comparatively, human melanoma cell lines were used, such as G-361 and A375, which were both supplied by ATCC (Manassas, VA, USA). Cells were maintained in MEM medium supplemented with 10% (*v*/*v*) heat-inactivated fetal bovine serum, 2 mM L-glutamine and 1% (*v*/*v*) streptomycin-penicillin solution. Cells were seeded on 24-well plates at the density of 0.5 × 10^5^ cells/well and allowed to attach to the surface of the subjected scaffolds for 24 h. After that, cells were cultured in a supplemented culture medium in a humidified atmosphere of 5% CO_2_ at 37 °C for 96 h while the culture medium was exchanged every 48 h. Differences in cell viability were assessed using the MTT assay.

### 2.9. Cell Viability Assay

MTT (5 mg/mL in 1 × PBS) was prepared in the respective culture medium (the final dilution, 1:10), in which 100 μL of the assay reagent was added to each well, and the cells were subsequently incubated for 3 h in a humidified atmosphere of 5% CO_2_ at 37 °C. The resultant formazan crystals were dissolved using 100 μL isopropanol/0.04 N HCl, absorbance was measured at *λ* = 595 nm using the BioTek ELx808™ microplate reader (BioTek Instruments, Inc., Winooski, VT, USA) and the results were normalized to the untreated control cells (Tissue Culture Plastic, TCP).

### 2.10. Statistical Analysis

Data were expressed as pooled means ± standard deviation (S.D.) of six independent experiments (*n* = 6). Statistically significant differences between results were determined by the univariate analysis of variance (ANOVA) or the Student’s *t*-test and appropriate post hoc analysis using GraphPad Prism 7.05 software (La Jolla, CA, USA). Differences at least at *p* < 0.05 were considered as statistically significant.

## 3. Results

### 3.1. Fourier Transform Infrared Spectroscopy–Attenuated Total Reflectance (FTIR-ATR)

**Figure 1:**
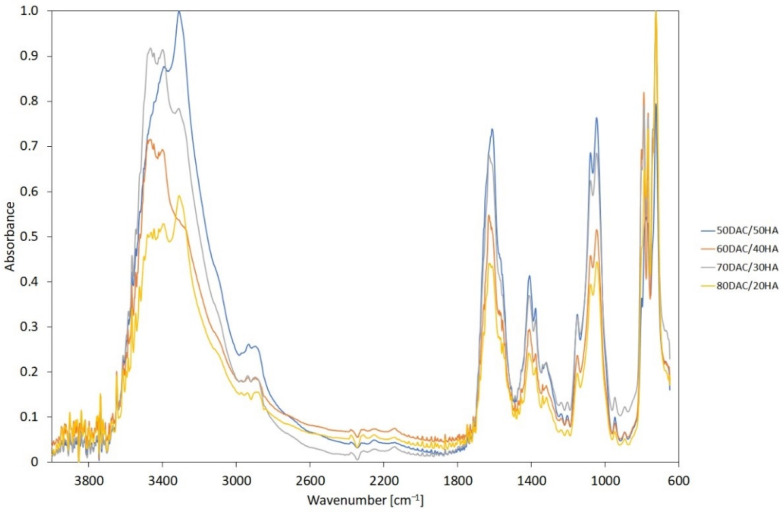
The FTIR-ATR spectra of dialdehyde chitosan/hyaluronic acid-based materials.

The FTIR-ATR analysis was carried out to detect the formation of characteristic bonds from hyaluronic acid, dialdehyde chitosan and bonds resulting from the mixing of these biopolymers. The FTIR-ATR spectra of obtained scaffolds based on dialdehyde chitosan/hyaluronic acid mixtures were recorded and shown in Figure 1.

### 3.2. Scanning Electron Microscopy (SEM)

**Figure 2:**
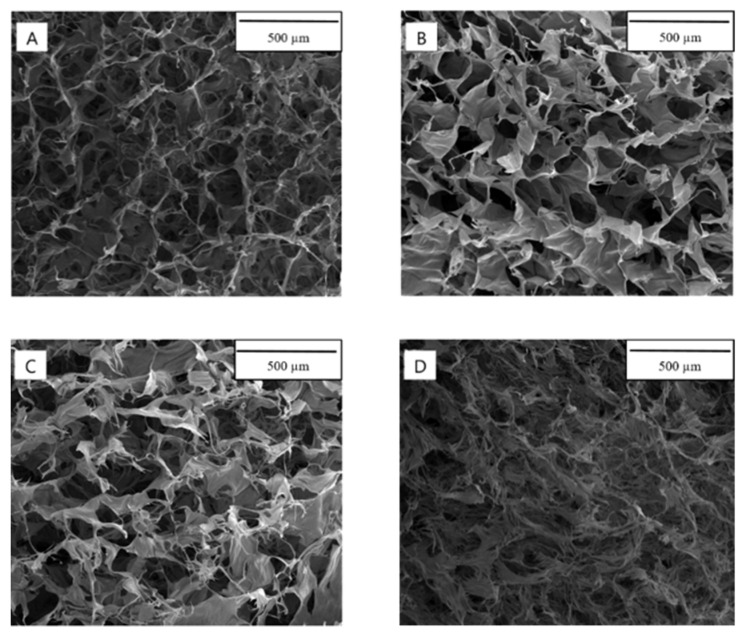
SEM images of scaffolds based on (**A**) 50DAC/50HA, (**B**) 60DAC/40HA, (**C**) 70DAC/30HA, (**D**) 80DAC/20HA (1 × 200).

Scanning electron microscopy images of dialdehyde chitosan/hyaluronic acid-based materials were presented in Figure 2. Each scaffold had a homogeneous structure and presented a porous structure with interconnected pores. They are essential to allow nutrients to flow inside the scaffold targeting cell proliferation within its entire volume. On the other side, changes in the diameter of the pores are not significantly dependent on the sample content.

### 3.3. Thermal Properties

The thermal properties of biopolymeric materials are crucial to be considered, as biopolymers have a low denaturation temperature. It limits their sterilization methods as thermal treatment cannot be used. Temperatures for maximum peaks were determined (Table 1). The first peak may be correlated to the elimination of water molecules present in the scaffold. Furthermore, two more regions in DTG curves may be distinguished. They can be assigned to the degradation of the polymeric structure of scaffold components. The highest T_max_ (2) and T_max_ (3) were noticed for scaffolds with the highest chitosan dialdehyde content. It can be assumed that a temperature lower than 130 °C is safe and does not cause the degradation of the scaffolds.

### 3.4. Mechanical Properties

**Figure 3:**
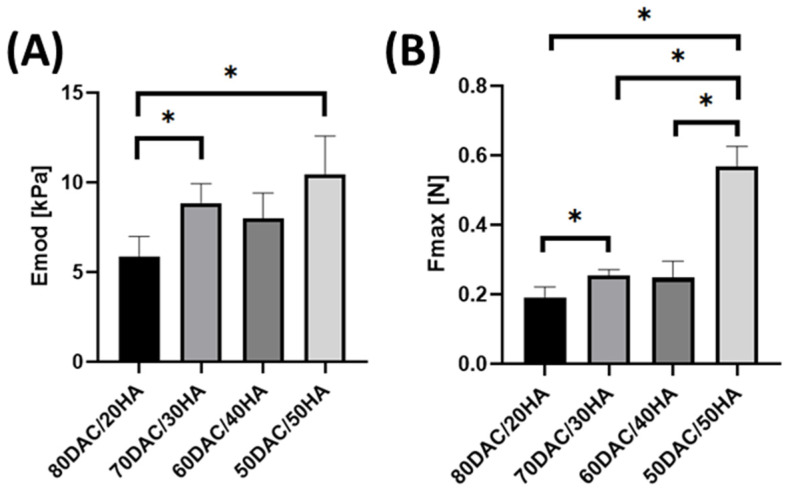

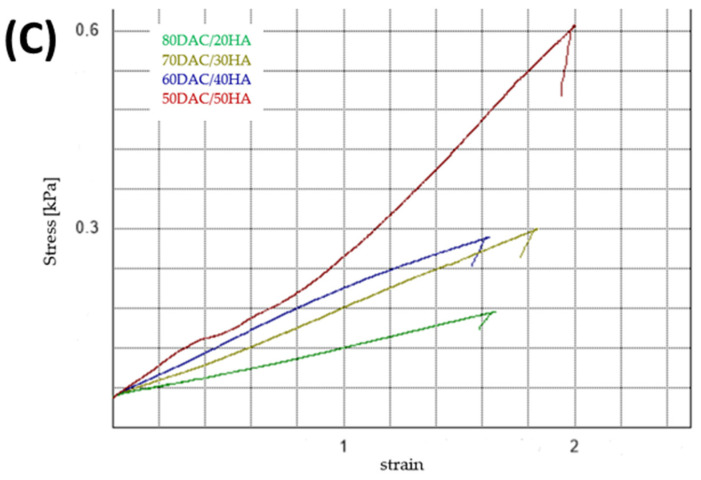
Mechanical parameters of dialdehyde chitosan/hyaluronic acid (DAC/HA) scaffolds: (**A**) compressive modulus (E_mod_), (**B**) maximum compressive force (F_max_), (**C**) stress strain curves (* significantly different, *p* < 0.05).

Assessment of mechanical parameters answers the question of whether material modifications are effective. In Figure 3, compressive modulus and maximum compressive force were shown. Mechanical parameters depend on the material composition (Figure 3). Higher hyaluronic acid content resulted in the improvement of compressive modulus (E_mod_) and maximum compressive force (F_max_). The highest parameters were noticed for scaffolds composed of 50DAC/50HA.

### 3.5. Density, Porosity and Water Content

In order to evaluate whether the obtained scaffolds are appropriate for tissue engineering applications, their density, porosity and water content were measured. The obtained results are shown in Table 2.

As it can be observed, the density of the studied scaffolds was in the range of 25.41 ± 2.20 mg/cm^3^ to 31.40 ± 2.31 mg/cm^3^. The highest density was observed for 70DAC/30HA scaffold. Porosity is a very important parameter in tissue engineering. The studied scaffolds were characterized by porosity higher than 80%, which is desirable in this field. The highest porosity was observed for the 80DAC/20HA scaffold, and the lowest for 60DAC/40HA, but there were no significant differences between the studied materials. The highest water content was found in scaffolds made of dialdehyde chitosan and hyaluronic acid in the proportion 50/50. Generally, water content should be higher when the content of hyaluronic acid in the material increases. This is because hyaluronic acid is strongly hygroscopic and has a high ability to absorb a large amount of water. As expected, in this study, one derogation was observed: the 60DAC/40HA material was characterized by a lower moisture content than the 70DAC/30HA scaffold.

### 3.6. Cellular Assessments Using Cutaneous Models

**Figure 4:**
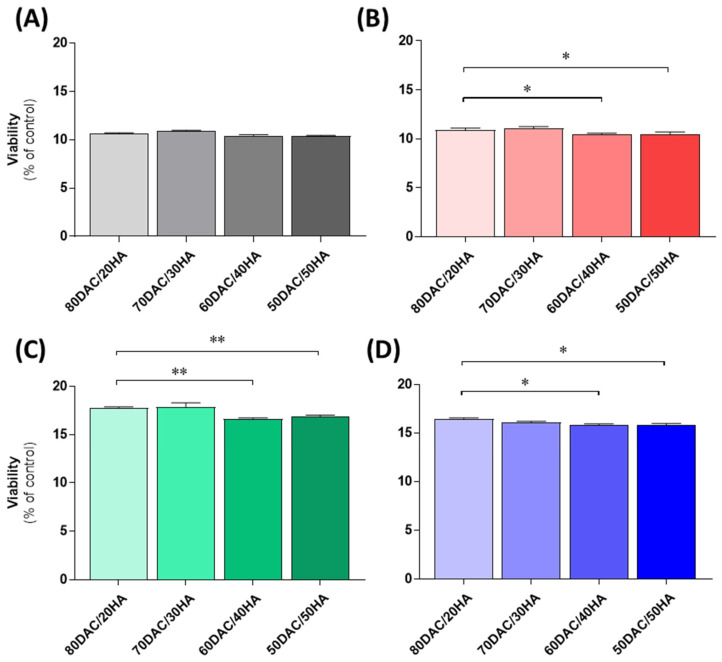
Human epidermal keratinocytes (NHEK) (**A**), dermal fibroblasts (NHDF) (**B**), as well as human melanoma cells (A375, (**C**) and G-361, (**D**)) were seeded on resultant scaffolds, cultured for 96 h, and viability was assessed using the MTT viability assay as described in the Materials and Methods. Data are presented as mean + S.D. (*n* = 6). Statistically significant differences were indicated accordingly as * *p* < 0.05, ** *p* < 0.01.

We performed the evaluation of cell proliferation in the presence of the examined scaffolds where significant differences were presented accordingly in Figure 4A–D. Among all the investigated cellular models, we noticed prominent differences between 80DAC/20HA and other compositions. A similar pattern of regulation was noticed within human epidermal keratinocytes, dermal fibroblasts and comparatively investigated melanoma models (A375 and G-361). The microscopic observation carried out during the experiment allowed us to observe that the cells had elongated shapes and were attached to the surface of the materials. No significant differences were noticed between other scaffolds, and this observation was visible in all the examined cell lines.

## 4. Discussion

3D materials with highly porous structures are desired candidates for tissue regeneration where significant enhancement of the nutrient maintenance for targeted cutaneous cells is required. We also noticed that the resultant materials kept their shapes and homogeneity. The FTIR analysis allows us to observe the presence of functional groups in the DAC/HA scaffolds as well as their shifts, which may indicate the hydrogen interactions (~1075 cm^−1^, C–O stretching vibrations peak), and the frequency of vibrations can depend on the strength of hydrogen bonds that stabilize the structure of scaffolds [20]. The characteristic bands of chitosan dialdehyde and hyaluronic acid were observed: NH– stretching vibration belongs to the amide A bond, near to 3310 cm^−1^ [21]; the amide I, II and III bands have been noticed around 1620 cm^−1^, 1575 cm^−1^ and 1205 cm^−1^, respectively [16,22]. Schiff’s base formation occurs between the free amino groups of hyaluronic acid and available aldehyde groups present in dialdehyde chitosan [20,23]. Taking into account the different weight compositions of scaffolds based on chitosan dialdehyde and hyaluronic acid, there are no significant differences in band locations, but some intensity changes can be observed. As it is seen in Figure 1, around 1613 cm^−1^, a sharp band is formed and it is derived from the carbonyl group. This band is a confirmation of the expected chitosan oxidation [14,16].

Higher chitosan dialdehyde content results in the increase of the maximum temperature of the thermal degradation process. It suggests that DAC presence improves the thermal properties of scaffolds. Hyaluronic acid is characterized as a polymer with low thermal stability [24]. However, mixing it with other biopolymers improves the thermal stability of the obtained material [25]. Following Miranda et al. [26], the scaffolds based on chitosan/hyaluronic acid mixtures are degraded at intermediate temperatures between 225 and 285 °C, which is similar to our results, where degradation was noticed between 230 and 305 °C.

Mechanical parameters measured for DAC/HA scaffolds were higher for scaffolds with higher content of hyaluronic acid. Correia et al. [27] studied chitosan/hyaluronic acid scaffolds with various amounts of hyaluronic acid (1, 5 and 10%). They reported that compressive modulus in dry and wet state increases with decreasing amount of hyaluronic acid in the material. It is the opposite relationship to the one discovered in our study, where we are using dialdehyde chitosan instead of chitosan in the material composition. In our opinion, it is related to the ability of hyaluronic acid to bind water molecules from the air, which stabilizes the material structure [28], and to a bigger amount of functional groups (aldehyde groups) in the dialdehyde chitosan, which are responsible for the greater reactivity of dialdehyde chitosan than chitosan [14,16].

The density of each type of scaffold was in the range of 28–31 mg/cm^3^. Higher hyaluronic acid content results in the decrease of porosity as more interactions occur between components. All the materials have been characterized by porosity higher than 80%, which is desirable in the biomedical field. Chanda et al. [29] studied chitosan/polycaprolactone-hyaluronic acid scaffolds, and they reported an increase in porosity after incorporating hyaluronic acid into chitosan/polycaprolactone scaffolds. Materials with the addition of hyaluronic acid were characterized by a porosity of about 90%, which is comparable with our results. The highest water content was noticed for the scaffold composition 50DAC/50HA, as hyaluronic acid is able to bind many water molecules due to its highly hydrophilic character.

Comparatively, we performed a cell viability assessment of the examined scaffolds [30] using epidermal keratinocytes, dermal fibroblasts and selected melanoma cell lines. Thus, we noticed slight but statistical differences between respective components as presented in Figure 4. Namely, it was visible that scaffolds containing 50DAC/50HA or 60DAC/40DA affected cell viability to a greater degree than those which contained 80DAC/20HA, indicating that an increasing amount of HA was the crucial factor. This pattern of regulation is in line with other physicochemical properties enclosed in this study. Nevertheless, further evaluations are highly essential to deepen and understand the correlation between cell proliferation and the composition of scaffolds targeted in wound healing.

## 5. Conclusions

Scaffolds based on dialdehyde chitosan and hyaluronic acid were obtained. They had a porous structure with interconnected pores. The thermal degradation of scaffolds was observed above 130 °C. Depending on the material composition, we observed the improvement of mechanical parameters with increasing hyaluronic acid content. The material composition did not affect the cells’ viability. The porosity of the material was around 90%, which allows us to classify it as highly porous and thereby makes it suitable for application in tissue engineering. Comparing the results for all the tested materials, it can be seen that the material composition has the greatest impact on the mechanical parameters. No significant differences were observed in the remaining analyses. Therefore, the 50/50 mixture of dialdehyde chitosan and hyaluronic acid is considered to be the best mixture because it has the highest mechanical parameters, with no deterioration of other parameters, including biological ones.

## Figures and Tables

**Table 1:** The results of DTA analysis with temperatures of maximum peaks.

Specimen	T_max_ (1) [°C]	T_max_ (2) [°C]	T_max_ (3) [°C]
80DAC/20HA	142.07	255.75	304.56
70DAC/30HA	133.33	236.20	270.76
60DAC/40HA	143.44	233.00	277.13
50DAC/50HA	163.93	238.71	289.31

**Table 2:** The results of density (d), porosity (ε) and water content of dialdehyde chitosan/hyaluronic acid scaffolds.

Specimen	d [mg/cm^3^]	ε [%]	Water Content [g/100 g]
80DAC/20HA	28.52 ± 7.86	91.93 ± 5.73	8.76 ± 0.70
70DAC/30HA	31.40 ± 2.31	89.49 ± 1.82	8.80 ± 1.66
60DAC/40HA	25.41 ± 2.20	89.06 ± 2.92	5.77 ± 2.09
50DAC/50HA	29.01 ± 4.23	89.94 ± 1.02	10.38 ± 1.42

## Data Availability

The data presented in this study are available on request from the corresponding author. The data are not publicly available due to project realization.
